# Author’s reply

**Published:** 2010

**Authors:** Arshad A. Bachh

**Affiliations:** *Department of Pulmonary Medicine, Mamata Medical College & Hospital, Khammam, Andhra Pradesh, India. E-mail: doc_arshad@yahoo.com*

Sir,

First I would really thank Dr. Vengattaraman[1] for showing keen interest in my article.[2] I would like to provide clarifications to the points raised for the benefit of all the readers of the article.

Query no 1 - Response:

The total number of cases diagnosed is 60 as noted in Table 2. Exclusive diagnosis mentioned in Table 4 implies the cases wherein bronchoscopy alone could make a definitive diagnosis possible, excluding those which were diagnosed on prebronchoscopic sputum culture. This exclusive diagnosis which would not have been possible without doing a bronchoscopy has been expressed as percentage viz 66% (40/60) and not as number of patients (ie 40), which the reader has not noted. Same thing is clearly mentioned in text para three on page 60.[2]

Query no 2 - Response:

Regarding the four cases which were sputum culture positive, it is infact, a little confusing as to why bronchial washings turned out to be negative in them. Inhibitory effect of topical xylocaine may have contributed to culture negativity in them, as each bacillus might have a different threshold for growth inhibition, and the amount of topical anesthetic actually reaching the bacilli might vary, although care was taken to use minimal topical anesthetic during the procedure. However, bronchoscopy still was useful in detecting non caseating granulomas in all these four cases out of which two turned out to be post bronchoscopy smear positive for AFB as well.[2]

Query no 3 - Response:

As all the cases were AFB smear negative, the risk of infection and hazard to bronchoscopist is minimal after using infection control measure as is clear from plenty of published literature on bronchoscopy in sputum smear negative pulmonary tuberculosis. Further, to get a *negative culture* report to exclude tuberculosis even by radiometric culture methods, (like BACTEC) it usually takes six weeks time, waiting for which would have delayed bronchoscopy and mitigated the early confirmation of tuberculosis thus defeating the very purpose of the study design, which was to assess the immediate diagnostic potential of a bronchoscopic procedure in sputum negative pulmonary tuberculosis (48.3% in the present study). Moreover, as there is no method to predict which smear negative patient will turn up to be culture positive in two weeks as suggested by the reader, it was not possible to exclude them initially.[2]
